# Evaluation of the performance of microprocessor-based colorimeter

**DOI:** 10.1155/S146392469200035X

**Published:** 1992

**Authors:** S. S. Randhawa, R. C. Gupta, A. K. Bhandari, P. S. Malhotra

**Affiliations:** Central Scientific Instruments Organisation Chandigarh 160020 India

## Abstract

Colorimetric estimations have an important role in quantitative studies. An inexpensive and portable microprocessor-based colorimeter developed by the authors is described in this paper. The colorimeter uses a light emitting diode as the light source; a pinphotodiode as the detector and an 8085A microprocessor. Blood urea, glucose, total protein, albumin and bilirubin from patient blood samples were analysed with the instrument and results obtained were compared with assays of the same blood using a Spectronic 21. A good correlation was found between the results from the two instruments.


					Journal of Automatic Chemistry, Vol. 14, No. 5 (September-October 1992), pp. 185-188
Evaluation of the performance of a
microprocessor-based colorimeter
S. S. Randhawa, R. C. Gupta, A. K. Bhandari and P. S.
Malhotra
Central Scientific Instruments Organisation, Chandigarh-160020, India
Colorimetric estimations have an important role in quantitative
studies. An inexpensive and portable microprocessor-based colori-
meter developed by the authors is described in this paper. The
colorimeter uses a light emitting diode as the light source; a pin-
photodiode as the detector and an 8085A microprocessor. Blood
urea, glucose, total protein, albumin and bilirubin from patient
blood samples were analysed with the instrument and results
obtained were compared with assays of the same blood using a
Spectronic 21. A good correlation was found between the results
from the two instruments.
Instrument
A block diagram of the instrument is shown in figure 1.
Light emitting diodes (LEDs) are used as the light source
because LEDs offer a fixed wavelength, narrow emission
profile and low power requirements. Because the band
width is narrow, filters are not required. LEDs with
emission peaks at 560 nm, 580 nm and 630 nm were
positioned on one side of a well containing the test
solution in a cuvette; a photodetector was placed on the
opposite side [1, 2]. The unit compares transmittance of
sample with a reference. Transmittance signals are fed
through the A/D converter to the microprocessor for
further processing for absorbance, and, finally, the
concentration of unknowns in the sample are displayed
and printed out.
Introduction
Estimating the amounts of urea, glucose, protein and
bilirubin in blood is important in the diagnosis of such
disorders as diabetes, kidney disease and liver malfunc-
tion. Developments in diagnostic techniques and elec-
tronic equipment have made it easier to carry out various
body function tests; this paper describes a system for the
analysis of blood biochemical parameters.
The system was designed to allow different modes of
operation. The first operating mode checks the 'zero'
position (calibration), and then autocalibrates to 100%
transmission (T) with distilled water at a particular wave
length. The next operating mode is to put the blank
reagent, as well as standard known concentration solu-
tion, in the cuvette and store the absorbance value in the
memory. The last operating mode is then used and the
results are displayed. A flow chart of the operation of the
system is given in figure 2.
COt40 ET ER
I
Figure 1. Block diagram ofmicroprocessor based colorimeter.
185
0142-0453/92 $3.00 ( 1992 Taylor & Francis Ltd.
S. S. Randhawa et al. Evaluation of the performance of a microprocessor-based colorimeter
AUTO
ZERO
SWITCH NO
NO
STORED THIs VALUE
AT M3 & M4 IN RAM
AND ADDED TO
ACTUAL
TRANSMISSION
YES
READ 14 BITS OF
A/D WITH POLARITY
AND OVER RANGE
DISPLAY ZERO (000.0)
THROUGH THE LED'S
IS
AUTO CAL
SWITCH
PRESSED
NO
YES
STORED THIS VALUE
AT MI M2 IN RAM
AND SUBTRACTED
FROM ACTUAL
TRANSMISSION
YES
READ 12 BITS OF
A/D VALUE
A/D VALUE
CORRECTED BY AUTO
ZERO VALUE
CALCULATE MUL.
FACTOR BY 100/AD
VALUE STORE AT M5
DISPLAY 100.0
THROUGH LED'S
IS
SC
SWITCH
PRESSED
YES
NO
Figure 2. Flowchart ofthe operation sequence.
Results and discussion
Photometric stability of the instrument was studied over
30 min with distilled water at 100% transmission (T)
stability was achieved after 10 min. Concentration
linearity was evaluated using a standard solution
prepared for each parameter.
Blood samples were taken from patients. Blood urea,
glucose, total protein, albumin and bilirubin were
assayed using the Thiosemicarbazide, Nelsonsomogi,
Biuret, Bromocresolgreen and Jenedrassik methods re-
spectively. Blood urea, total protein, glucose were
assayed at 560 nm, albumin was measured at 630 nm and
bilirubin at 583 nm using different light emitting diodes.
186
S. S. Randhawa et al. Evaluation of the performance of a microprocessor-based colorimeter
LOAD STANDARD
CONCENTRATION AND
STORE IT IN SOME
MEMORY LOCATION
H NO
YES
READ TRANSMISSION
FROM A/D AND IS
CORRECTED BY AUTO
ZERO VALUE AND
AUTO CAL. MULTIPLYING
FACTOR AND
DISPLAY TRANSMISSION
THIS TRANSMISSION
IS CONVERTED TO
SOD WITH HELP OF
LOOK UP TABLE AND
STORE AT SOME
MEMORY LOCATION
ABOVE THREE STEPS
ARE REPEATED FOR
CALCULATING ROD
AND OPTICAL
DENSITIES (O.D.) OF
UNKNOWN SAMPLES
SOLVE EQUATION
FOR CONCENTRATION
OF UNKNOWN SAMPLE
OD.U-OD.R
CU XCON.
OD.S-OD.R
OF STANDARD SAMPLE
SAMPLE CONCENTRATION
THROUGH THE LED'S
Figure 2 (continued).
Table compares the results obtained with the authors
instrument with those obtained with a Spectronic 21.
Good correlation was found between the two sets of
results. The following regression equations and coef-
ficients of correlation were observed:
Urea
Glucose
Total protein
Albumin
Bilirubin
R2
0"998;
R 0"996;
R2
0"958;
R 0"969;
R 0"999;
y= 0-994x+ 0"569 (1).
y 0"956x + 5"832 (2).
y 0"953x + 0359 (3).
y 0-971x + 0"019 (4).
y= l'016x+ 0"809 (5).
Table 1. Regression analysis.
No. of Mean value
obser- CSIO Mean value
Parameters vations Colorimeter Spectronic 21 e
X Co-
efficient Regression line
Urea 50 67"576 67"760
Glucose 22 152",99 152"22
Total protein 24 5"84 5"92
Bilirubin 11 8"9 9"0
Albumin 20 4-18 4"04
0"998
0.996
0.958
0.999
0"969
0.994
0.956
0.953
1.016
0.971
y 0.994x + 0.569
y 0.953x + 5"832
y 0.953x + 0.359
y= 1.016x+ 0"809
y 0.971x + 0.019
187
S. S. Randhawa et al. Evaluation of the performance of a microprocessor-based colorimeter
26
24
22
20
6 14
12
8
2 4 6 8 10 12 14 16 18 20 22 24 26
Microprocessor Based Colorimeter (mg%)
300
280
260
240
220
200
180
a6o
140
120
100
80
80 100 120 140 160 180 200 220 240 260 280 300
Microprocessor Based Colorimeter (mg%)
Figure 3. Comparison of results for bilirubin determined on the
Spectronic-21 (y-axis) and the microprocessor-based colorimeter
(x-axis).
Figure 4. Comparison of results for glucose determined on the
Spectronic-21 (y-axis) and the microprocessor-based colorimeter
(x-axis).
The values of serum bilirubin and glucose assays
obtained with microprocessor-based colorimeter were
plotted against those obtained with the Spectronic and
are shown in figures 3 and 4.
Conclusion
The results indicate that the instrument has a precision
and reproducibility which is at least as good as a
commercial instrument. In addition, the system has a
number of attractive features of the system: for example
no filter is used, concentrations are printed out, and there
is no possibility of confusion over the sample number
being tested.
References
1. ROSF., A. W. and STEADMAN, J. W., Clinical Chemistry, 20
(1974), 613-614.
2. JAFFAR, M. and ZAHID, Q., Journal of Chemical Education, 85
(1988), 1099-1100.
188

				

